# Probiotics/Synbiotics to Reduce Infectious Complications after Colorectal Surgery: A Systematic Review and Meta-Analysis of Randomised Controlled Trials

**DOI:** 10.3390/nu14153066

**Published:** 2022-07-26

**Authors:** Julie Veziant, Mathilde Bonnet, Bob V. Occean, Chadly Dziri, Bruno Pereira, Karem Slim

**Affiliations:** 1 Department of Digestive and Oncological Surgery, University Hospital Lille, 59000 Lille, France; julieveziant@gmail.com; 2 The Francophone Group for Enhanced Recovery after Surgery, GRACE, 63110 Beaumont, France; 3 M2iSH UMR 1071 Inserm/Clermont Auvergne University, USC-INRAE 2018, CRNH, 63000 Clermont-Ferrand, France; mathilde.bonnet@uca.fr; 4 Department of Statistics, University Hospital, 30000 Nîmes, France; bob.occean@chu-nimes.fr; 5 Honoris Medical Simulation Center, Tunis 1000, Tunisia; cdziri@tn.honoris.net; 6 Department of Statistics, University Hospital CHU Clermont-Ferrand, 63000 Clermont-Ferrand, France; bpereira@chu-clermontferrand.fr; 7 Department of Digestive Surgery, University Hospital CHU Clermont-Ferrand, 63000 Clermont-Ferrand, France

**Keywords:** probiotics, synbiotics, colorectal, surgery, postoperative complications, meta-analysis

## Abstract

Aim: The aims of this systematic review and meta-analysis were to assess to what extent probiotics/synbiotics reduce infectious complications after colorectal surgery and whether probiotics or synbiotics should be considered as perioperative measures preventing or reducing infectious complications after CRS and should be included in enhanced recovery programmes (ERP). Secondary aims were to answer practical questions precisely on the best formulation and the type and timing of probiotics or synbiotics in CRS. Method: This systematic review and quantitative meta-analysis were conducted in accordance with PRISMA 2020 guidelines. Inclusion criteria were randomised trials comparing perioperative probiotics/synbiotics with a placebo or standard care in elective colorectal surgery. Exclusion criteria were non-randomised trials. Overall infectious complications and surgical site infections (SSIs including both deep abdominal infections and wound (skin or under the skin) infections) were the primary outcomes. Secondary outcomes were pulmonary and urinary infections, wound infections, and anastomotic leaks. The databases consulted were Medline, Cochrane Database of Systematic Reviews, Scopus, and Clinical Trials Register. Risk of bias was assessed according to the GRADE approach. The analysis calculated the random effects estimates risk ratio (RR) for each outcome. Results: 21 trials were included; 15 evaluated probiotics, and 6 evaluated synbiotics. There were significantly fewer infectious complications (risk ratio (RR) 0.59 [0.47–0.75], I^2^ = 15%) and fewer SSI (RR 0.70 [0.52–0.95], I^2^ = 0%) in the probiotic or synbiotic group. There were also significantly fewer pulmonary infections (RR 0.35 [0.20–0.63]) and urinary infections RR 0.41 [0.19–0.87]) as opposed to anastomotic leaks (RR 0.83 [0.47–1.48]) and wound infections (RR 0.74 [0.53–1.03]). Sensitivity analyses showed no significant difference between probiotics and synbiotics in reducing postoperative infections (RR 0.55 [0.42–0.73] versus RR 0.69 [0.42–1.13], *p* = 0.46). Conclusions: Based on the finding of this study, probiotics/synbiotics reduce infectious complications after colorectal surgery. The effect size was more pronounced for pulmonary and urinary infections. From a practical aspect, some of the questions related to formulations and duration of probiotics or synbiotics need to be answered before including them definitively in enhanced recovery after colorectal surgery programmes.

## 1. Introduction

Despite significant improvement in surgical care, colorectal surgery (CRS) is still associated with significant postoperative infectious complications, including surgical site infection, and are the most common health-care-associated infections [1]. Numerous medical measures are advocated to reduce the incidence of such complications [2], both preoperatively (smoking cessation, nutritional status improvement, colonic decontamination using oral antibiotics, intravenous antibiotics and intraoperatively (hypothermia prevention, oxygen supplementation, skin preparation, abdominal wall protection, and minimally invasive surgical approaches) [3].

Besides the ongoing debate on the role of mechanical bowel preparation associated with oral antibiotics [4], recent evidence-based findings suggest that oral antibiotics (OABs) do reduce the incidence of surgical-site infections without mechanical bowel preparation [5].

In contrast to the abundant literature on bowel preparation, there are relatively fewer meta-analyses addressing the role of perioperative probiotics or synbiotics for the reduction in infectious complications after CRS. These dietary supplements comprise three formulations. According to the ISAPP (International Scientific Association for Probiotics and Prebiotics) consensus statement [6], probiotics are live microorganisms that, when administered in adequate amounts, confer a health benefit to the host. A synbiotic is a combination of a probiotic and a prebiotic (an indigestible food ingredient that stimulates the activity of some bacteria) claimed to be more efficient than the probiotic alone. Most probiotics or synbiotics include species (or strains) of *Bifidobacterium*, *Streptococcus*, *Lactobacillus*, or *Propionibacterium*. Early reports suggested that probiotics or synbiotics had a positive effect on gut microflora, intestinal structure, and function, and on local immune response. Thus, they can be useful preoperatively as an adjunct to bowel preparation (by reducing the related overgrowth of pathogenic bacteria) and perioperatively to reduce infectious complications (by preserving the mucosal gut barrier) [7]. However, the conflicting results of published trials suggest that intrinsic mechanisms are complex and elusive, and the heterogeneity of study doses, timing, duration, and number and types of strains preclude any definite conclusions [8].

Eight meta-analyses focusing on the role of probiotics or synbiotics in CRS were published [9,10,11,12,13,14,15,16] before our meta-analysis. We first set out to make an umbrella review of these meta-analyses (CRD42022304466). However, the low quality of most of the meta-analyses and their lack of comprehensiveness or reported relevant outcomes convinced us that an umbrella review would not answer our questions. We therefore continued the systematic review already started (CRD42020220290) rather than attempt an umbrella review [17]. The first aim of this systematic review and meta-analysis was to assess, by pooling the data of published randomised trials, whether probiotics or synbiotics are efficacious and so should be considered as perioperative measures preventing or reducing infectious complications after CRS and should be included in enhanced recovery programmes (ERP). Secondary aims were to answer practical questions precisely on the best formulation and the type and timing of probiotics or synbiotics in CRS.

## 2. Methods

### 2.1. Review Design

This systematic review and meta-analysis followed the Preferred Reporting Items for Systematic Reviews and Meta-Analyses PRISMA-2020 guidelines [18]. The review was registered in the PROSPERO-Register (CRD42020220290).

### 2.2. Search Strategy

An electronic search was conducted up to 14 February 2022 in the following databases: Medline, PICO (Patient, Intervention, Comparison, Outcome), Cochrane Database of Systematic Reviews, Scopus, and Clinical Trials Register. The references listed in each retrieved article were manually searched. The MeSH terms were: (probiotics) OR (synbiotics) AND (surgery) AND (colorectal). Selected publication languages were: English, French.

### 2.3. Inclusion Criteria

We included randomised controlled trials (RCTs) evaluating probiotics/synbiotics (Pro/Syn) in CRS. Congress abstracts were not included. The comparators considered were: probiotic or synbiotic vs. placebo or standard care.

### 2.4. Data Extraction

The titles and abstracts of all the identified reviews were screened by two independent assessors (JV, KS) against the inclusion and exclusion criteria. Any disagreement was resolved by consensus. The full texts of relevant RCTs were assessed independently. The selection/exclusion process is summarised in a PRISMA 2020 flow diagram [18].

We extracted a dataset from each included RCT: first author, year of publication, country, number of patients, formulation (prob or synb), pre- and/or postoperative, strains, main outcomes, competing interests of the authors, and comments.

Overall infectious complications and surgical site infections (SSIs including both deep abdominal infections and wound (skin or under the skin) infections) were considered as the primary outcomes. Anastomotic leaks, wound infections, urinary infections, pulmonary infections were the secondary outcomes.

Data were extracted by two independent assessors (JV, KS). Any discrepancy was resolved by consensus after checking the results.

### 2.5. Assessment of Methodological Quality

The methodological quality of all the included RCTs was assessed using the Jadad scale [19]. A score of 3–5 indicated a RCT of acceptable quality, and a score of 1–2 indicated a RCT of poor quality.

### 2.6. Subgroups Analyses

Several practical subgroup analyses were performed: probiotics vs. synbiotics, multistrains (≥3 agents) vs. non-multistrains, preoperative versus perioperative vs. postoperative, control group placebo vs. standard care, quality of RCTs, competing interests of authors, and ethnicity.

### 2.7. Certainty of Evidence Analyses

The risk of bias was evaluated using the Cochrane risk assessment tool (Cochrane Handbook, version 6.1, University of Bristol, Bristol, UK). The five assessed domains were: randomization process, deviations from intended intervention, missing outcome data, measurement of the outcome, and selection of the reported result. The risk of bias for each study was evaluated as low or high or unclear. Certainty of the evidence was assessed using the GRADE approach [20] in a “Summary of Findings” table by calculating the absolute and relative risks, and ranking the quality of evidence based on the risks of bias and publication bias, heterogeneity, and precision. Quality of evidence was rated high if further research is deemed unlikely to change confidence in the estimated effect, moderate if further research is deemed likely to have a significant impact on confidence in the estimated effect and could change the estimate, low if further research is deemed very likely to have a significant impact on confidence in the estimated effect and likely to change the estimate, and very low if the estimated effect is deemed very uncertain.

### 2.8. Data Synthesis and Statistical Analysis

This meta-analysis was performed combining the results of the reported risk ratio in the selected studies. Binary outcomes data from these studies were extracted when available. The analysis calculated the random effects estimates risk ratio (RR) for each outcome listed above. The inverse variance weighting was used for pooling. The iterative Paule–Mandel method was used to estimate between-study variance [21]. Heterogeneity between studies was explored using Cochran’s Q statistic, Higgin’s and Thompson’s percentage of variability I^2^ statistic to estimate the percentage of total variation across studies arising from heterogeneity rather than chance [22]. Heterogeneity was rated low, moderate, or substantial when the value of I^2^ was less than 25%, 50%, or greater than or equal to 75%, respectively. When there was evidence of heterogeneity (I^2^ ≥ 50 or clearly identified reason), a sensitivity analysis without the concerned trials was performed. The same methods were followed for the subgroup analyses. Publication bias was evaluated using a funnel plot. Statistical analysis was performed using the General Package for Meta-Analysis “meta” Version 4.9–1 with R software version 3.5.1 [23].

## 3. Results

### 3.1. Protocol Deviations

There was no discernible protocol violation, except that the meta-analysis took longer than expected due to the temporary unavailability of some team members.

### 3.2. Search Results and Trials’ Characteristics

**Figure 1:**
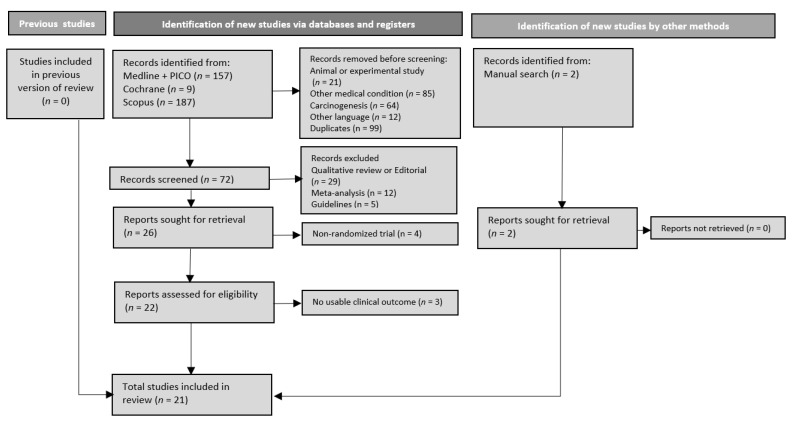
Flow diagram of the search strategy according to PRISMA 2020.

The flow diagram (Figure 1) of trials shows the inclusion/exclusion processes. Twenty-one RCTs were eventually included in the synthesis [24,25,26,27,28,29,30,31,32,33,34,35,36,37,38,39,40,41,42,43,44]. Table 1 summarises the characteristics of included RCTs. Briefly, most trials were published in the last decade (2010–2020), and more than half were from Asia. The number of patients included in these RCTs ranges from 33 to 362.

A total of 6 RCTs evaluated synbiotics [24,25,30,34,38,40], and 15 evaluated probiotics [26,27,28,29,31,32,33,35,36,37,39,41,42,43,44]. The control group received a placebo (*n* = 14) [25,26,27,28,29,32,36,37,38,39,40,42,43,44] or standard care (*n* = 7) [24,30,31,33,34,35,41]. A total of 8 RCTs involved a preoperative timing for probiotic or synbiotic [24,25,28,30,33,36,39,40], 10 both pre- and postoperative timing [26,27,29,31,34,35,37,38,43,44], and 3 a postoperative timing [32,41,42]. Duration of probiotic or synbiotic use ranged from 3 to 14 days preoperatively, and from 2 to 21 days postoperatively. In one RCT [41], the patients had to take probiotics for one year postoperatively. In the treated groups and in equal proportions, one third of RCTs used three strains (multistrains) [24,28,32,37,39,41,44]; one third used two strains [26,29,34,36,38,40,43]; and one third used one strain [25,27,30,31,33,35,42]. Details of the strains are summarised in Table 2.

A total of 6 RCTs showed that probiotics or synbiotics decreased postoperative infectious complications [26,28,29,32,38,40], while 14 RCTs showed no effect on postoperative infectious complications. In one trial [44], data were insufficient.

Finally, regarding competing interests, six teams declared they had competing interests [25,26,34,36,40,43]; nine declared they had no competing interests [24,27,29,32,37,39,41,42,44]; and six made no declaration [28,30,31,33,35,38].

### 3.3. Overall Results

A total of 1961 patients were included in the meta-analysis with 973 in the probiotic or synbiotic group and 988 in the control group (whether placebo or standard care).

#### 3.3.1. Primary Outcomes

**Figure 2:**
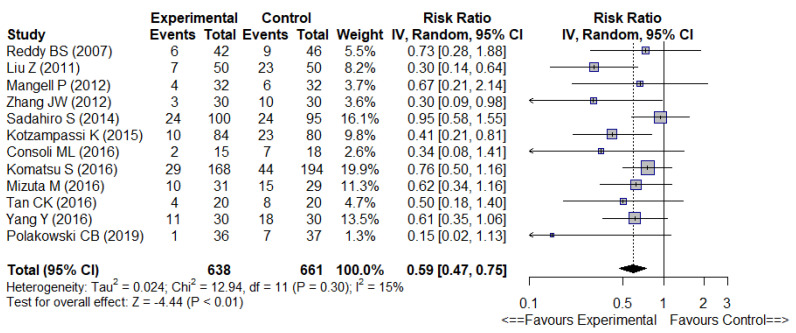
Forest plot of overall postoperative infectious complications [24,26,27,28,31,32,33,34,35,36,37,40].

**Figure 3:**
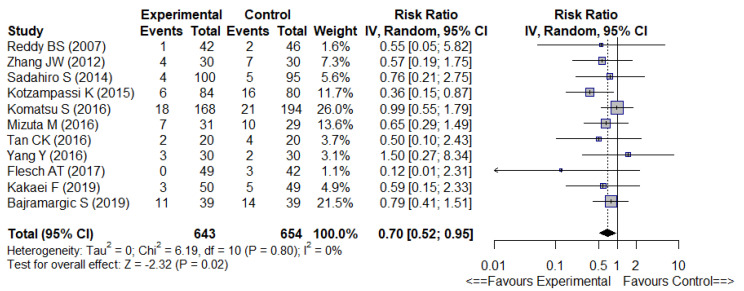
Forest plot of surgical site infections [24,28,31,32,34,35,36,38,39,41].

The meta-analysis (Figure 2) showed overall significantly fewer infectious complications (12 trials, RR 0.59 [0.47–0.75], *p* < 0.01) with low heterogeneity (I^2^ = 15%) and significantly fewer SSI (11 trials, RR 0.70 [0.52–0.95], *p* = 0.02) with no heterogeneity (I^2^ = 0%), in the probiotic or synbiotic group (Figure 3).

#### 3.3.2. Secondary Outcomes

There were significantly fewer pulmonary infections (10 trials, RR 0.35 [0.20–0.63], *p* < 0.01) with no heterogeneity (I^2^ = 0%), and significantly fewer urinary infections (6 trials, 0.41 [0.19–0.87], *p* = 0.02) with no heterogeneity (I^2^ = 0%) (Supplementary Online Data, Figures S1 and S2).

By contrast, the differences were not significant between the groups for anastomotic leaks (11 trials, RR 0.83 [0.47–1.48], *p* = 0.53, I^2^ = 29%) and wound infections (11 trials, RR 0.74 [0.53–1.03], *p* = 0.08) with no heterogeneity (I^2^ = 0%) (Figures S3 and S4).

### 3.4. Sensitivity (Subgroup) Analyses

We included studies reporting outcomes considered (primary and secondary). In some cases, some data were missing for some outcomes which explains the differences in the number of studies included in subgroup analyses.

#### 3.4.1. Probiotics versus Synbiotics

In total, there were 523 patients in the Synb group and 776 patients in the Prob group.

**Figure 4:**
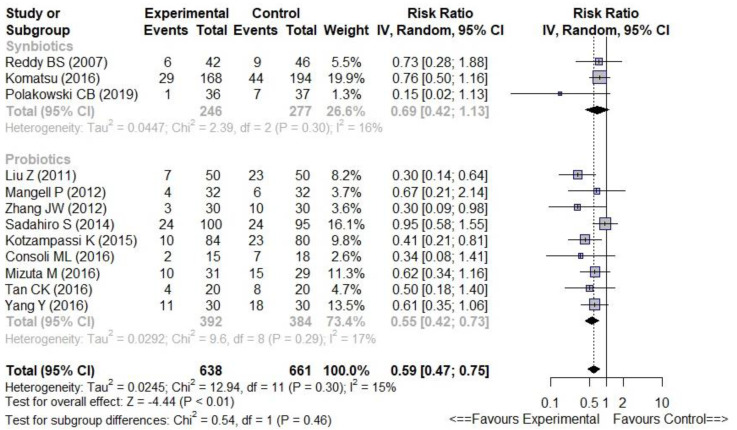
Forest plot of probiotics [26,27,28,31,32,33,35,36,37] versus synbiotics [24,34,40]. Outcome = overall infectious complications.

Since the main outcome (overall infectious complications) was considered in this subgroup analysis, only three trials (reporting this outcome) were included. There were fewer overall infectious complications (9 trials, RR 0.55 [0.42–0.73], I^2^ = 17%) (Figure 4) and fewer SSIs (8 trials, RR 0.63 [0.44–0.91], I^2^ = 0%) in the Prob subgroup (Figure S5) compared with controls. By contrast, there were no significant differences between the Synb subgroup and controls for overall infectious complications and SSIs (3 trials, RR 0.69 [0.42–1.13], I^2^ = 16% and RR 0.87 [0.47–1.60], I^2^ = 2%, respectively) (Figure 4 and Figure S5). Nevertheless, the comparison between the respective effects of probiotics and synbiotics showed no significant difference (*p* = 0.46, Figure 4).

#### 3.4.2. Multistrain vs. Non-Multistrain Formulations

**Figure 5:**
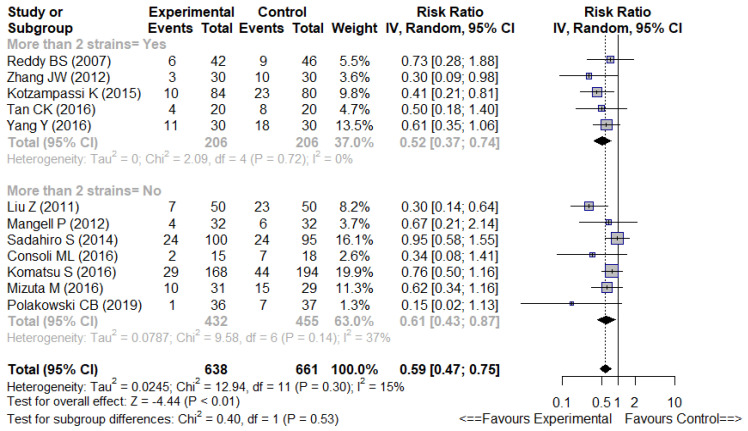
Forest plot of multistrains [7,24,28,32,36] versus non-multistrains [26,27,31,33,34,35,40] probiotic or synbiotic. Outcome = overall infectious complications.

There were no significant differences for overall infectious complications whether multistrain formulations (more than 2 strains) or non-multistrain ones were used (5 trials RR 0.52 [0.37–0.74], I^2^ = 0% and 7 trials RR 0.61 [0.43–0.87], I^2^ = 37%, respectively) (Figure 5).

#### 3.4.3. Preoperative vs. Perioperative vs. Postoperative

**Figure 6:**
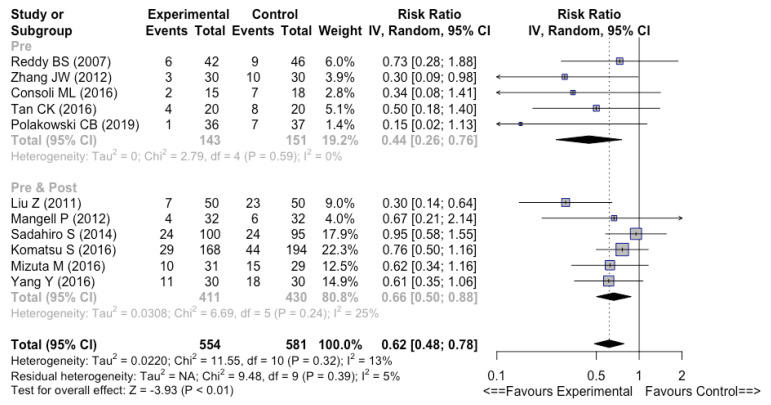
Forest plot of the timing of perioperative [26,27,31,34,35,37] versus preoperative [24,28,33,36,40] use of probiotics or synbiotics. Outcome = overall infectious complications.

There were no significant differences whether probiotics or synbiotics were used preoperatively or perioperatively (i.e., pre- and postoperatively) regarding overall infectious complications (RR 0.44 [0.26–0.76], I^2^ = 0% versus RR 0.66 [0.50–0.88], I^2^ = 25%, respectively) (Figure 6). In the trial published by Kotzampassi K et al. [32], the protocol involved, in contrast with other pre- and postoperative treatments [26,27,31,34,35,37], the administration of probiotics on the day of surgery. Hence, we did not consider it as pre- and postoperative treatment.

#### 3.4.4. Placebo vs. Standard Care Controls

The effect sizes were significantly higher when a placebo was used compared with standard care controls for both overall infectious complications (RR 0.45 [0.33–0.62], I^2^ = 0% versus RR 0.76 [0.58–0.99], I^2^ = 0%, respectively). However, for SSI, the difference was not statistically significant (RR 0.82 [0.57–1.18, I^2^ = 0%) (Figures S6 and S7).

#### 3.4.5. Quality of RCTs

Excluding the trials with a Jadad score of <3 did not modify the effect size regarding overall infectious complications (RR 0.56 [0.44–0.70], I^2^ = 0% versus RR 0.59 [0.47–0.75], I^2^ = 15% for overall results) (Figure S8).

#### 3.4.6. Ethnicity

There was no significant difference between the groups whether the RCTs were conducted in Asia or in the West for overall infectious complications (RR 0.62 [0.46–0.83], I^2^ = 31% versus RR 0.47 [0.30–0.75], I^2^ = 0%, respectively) (Figure S9).

#### 3.4.7. Competing Interests

There was no difference for overall infectious complications according to whether the authors declared they had competing interests or not (RR 0.47 [0.25–0.87], *p* = 0.10, I^2^ = 52% versus RR 0.56 [0.39–0.82], *p* = 0.74, I^2^ = 0%) (Figure S10).

### 3.5. Bias of Publication

**Figure 7:**
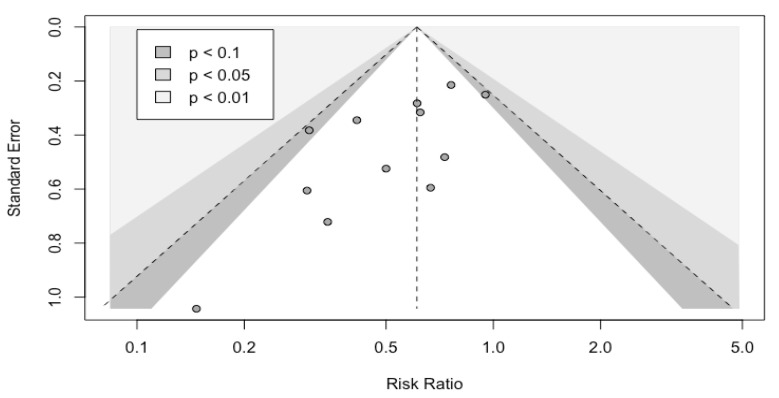
Funnel plot. Outcome = overall infectious complications.

The funnel plot (Figure 7) did not show a significant asymmetry.

### 3.6. Certainty of the Evidence

**Figure 8:**
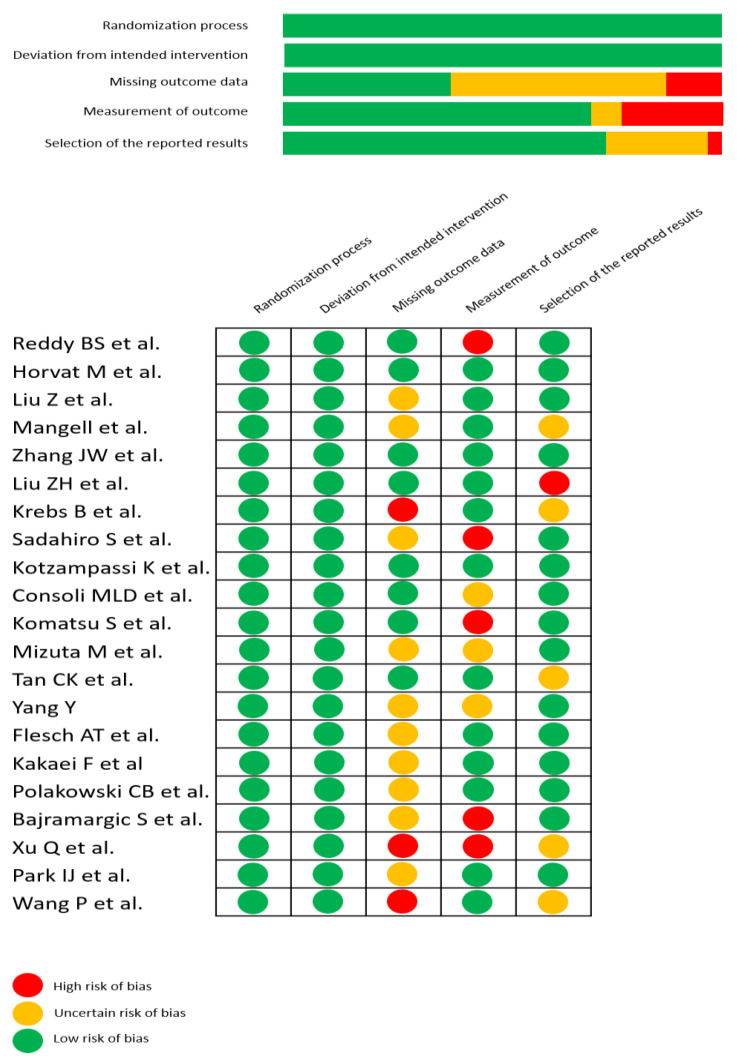
Certainty of evidence according to GRADE [24,25,26,27,28,29,30,31,32,33,34,35,36,37,38,39,40,41,42,43,44].

Certainty of the evidence according to GRADE [20] is summarised in Figure 8 and Table 3.

## 4. Discussion

To our knowledge, this study is the largest meta-analysis focusing on the role of probiotics or synbiotics in colorectal surgery. We addressed some clinical questions not answered by the previous meta-analyses [9,10,11,12,13,14,15,16]. Table 4 summarises the data of previously published meta-analyses with their main results. This meta-analysis has the advantage of going beyond the overall results and addressing practical questions through sub-group analyses. We confirmed the favourable effect of probiotics or synbiotics on overall postoperative infectious complications, but the effect size was lower than that reported in a previous similar meta-analysis [16] regarding overall infectious complications and SSIs. This discrepancy is probably due to the different inclusion criteria used in our meta-analysis (no Chinese RCTs but 9 further RCTs) and in the meta-analysis from Zeng et al. [16] (including several Chinese RCTs). On the other hand, our meta-analysis showed that probiotics were more effective than synbiotics in reducing postoperative infections after colorectal surgery. This finding is consistent with those reported by Zeng et al. [16] (associated with substantial heterogeneity in the synbiotic-subgroup I^2^ = 77%). This is counter-intuitive, especially as we can expect that prebiotics and probiotics (defining synbiotics) produce synergistic effects. A further meta-analysis published by Chen et al. [45] after the completion of our own meta-analysis included fewer (*n* = 14) trials than our meta-analysis and showed quite similar results. One meta-analysis [46] showed similar outcomes whatever the treatment used with an apparent effect of synbiotics. However, it included several abdominal surgeries and did not involve a colorectal subgroup analysis. The respective effectiveness of probiotics and synbiotics is not well explored in the literature. To the best of our knowledge, there has been no randomised trial comparing probiotics with synbiotics. Such studies are needed as their findings affect our daily practice. Figure 4 analysing probiotic and synbiotic subgroups separately suggests at first sight that the effect size increased (with a narrow confidence interval) in the probiotic group as opposed to the synbiotic group. However, we think that the lack of a significant effect of synbiotics is probably due the small number of trials included (*n* = 3), since the actual comparison between the probiotics and synbiotics effect showed no significant difference (*p* = 0.46, Figure 4).

We were unable to answer the question of the timing of treatment, since both preoperative and perioperative prescriptions are efficacious in reducing overall infectious complications. We were also unable to answer the question of the best strain formulations. Thus, it is difficult to determine which (and how many) strains are to be recommended in the formulations of probiotics. Table 2 shows that the most widely used strains are Lactobacillus and Bifidobacterium (in association in some trials with Streptococcus or Enterococcus). The cross-reference between Figure 4 and Table 2 may suggest that the best formulation is Bifidobacteria plus Lactobacillus, but this remains a mere hypothesis in the absence of double-blind randomised trials specifically assessing the formulation of probiotics. On the other hand, the role of yeasts used by some authors [32,33] needs to be assessed in future studies.

The subgroup analyses based on competing interests or ethnicity demonstrated that the efficacy of probiotics or synbiotics was not altered whether there were competing interests or not and whether the studies were conducted in Asia or in the West.

Some particular findings of our meta-analysis also deserve comment: the higher effect size of probiotics for “non-surgical” infectious complications, namely pulmonary and urinary infections. The size effects for these infections (RR = 0.35 for pulmonary infections and 0.41 for urinary infections, with no heterogeneity) were twice those for SSIs (RR = 0.70) or wound infections (RR = 0.74), or anastomotic leaks (RR = 0.83). In other words, even though probiotics do reduce SSI after colorectal surgery, their benefits are most obvious for “non-surgical” infectious complications, i.e., pulmonary and urinary infections. This finding was made by Liu et al. [11] in a previous meta-analysis, as shown in Table 4. Current literature data help us to formulate some hypotheses to explain this difference in effect size between “non-surgical” and “surgical” complications. There is accumulating evidence suggesting the influence of gut microbiota on lung immunity referred to as the gut-lung axis [47]. On the other hand, recent preliminary reports also suggested the role of probiotics in preventing or treating urinary tract infections [48].

These data recall what is observed in enhanced recovery programmes, where the major benefits are concerns so-called “non-surgical” morbidity [49].

In our opinion, there is now enough evidence to include probiotics in enhanced recovery (after colorectal surgery) programmes. Furthermore, since enhanced recovery programmes reduce postoperative ileus [50], we can assume that the efficacy of probiotics would be further improved in this setting.

The strengths of this meta-analysis are that it is based on mostly well-conducted randomised trials (15 out of 21 RCTs—72% have a Jadad scale >3) and the lack of discernible heterogeneity or publication bias. The meta-analysis updates the data published during the last decade and answers some of our practical questions (such as probiotics vs. synbiotics) in colorectal surgery.

The main limitation is related to the diversity of formulations, dosages, and durations of treatment of probiotics or synbiotics in the studies. This prevents us from determining the best practical approach.

## 5. Conclusions

This meta-analysis confirmed the efficacy of probiotics or synbiotics in reducing infectious complications after colorectal surgery. It suggests that the effect size is higher for “non-surgical” infectious complications. However, from a practical aspect, the timing and the formulation of probiotics or synbiotics need further studies before formally including probiotics or synbiotics in enhanced recovery (after colorectal surgery) programmes.

## Figures and Tables

**Table 1:** Characteristics of the included randomised trials [24,25,26,27,28,29,30,31,32,33,34,35,36,37,38,39,40,41,42,43,44].

First Author (Year)	Country	Number of Patients	Formulation	Control	Timing	Duration	Strains	Effect on Infectious Complications	Competing Interests	Jadad Scale
Reddy BS (2007) [24]	Denmark	88	Synbiot	SC	Pre	NA	L + B + S	No effect	No	3
Horvat M (2010) [25]	Slovenia	40	Synbiot	Placebo	Pre	NA	L	No effect	Yes	5
Liu Z (2011) [26]	China	100	Probiot	Placebo	Pre and Post	6d & 10d	L + B	Decreased infectious complications	Yes	5
Mangell P (2012) [27]	Sweden	64	Probiot	Placebo	Pre and Post	8d & 5d	L	No effect	No	4
Zhang JW (2012) [28]	China	60	Probiot	Placebo	Pre	3d	L + B + E	Decreased infectious complications	Not declared	4
Liu ZH (2013) [29]	China	150	Probiot	Placebo	Pre and Post	6d & 10d	L + B	Decreased infectious complications	No	5
Krebs B (2013) [30]	Slovenia	34	Synbiot	SC	Pre	3d	L	No effect	Not declared	4
Sadahiro S (2014) [31]	Japan	195	Probiot	SC	Pre and Post	7d & 5d	B	No effect	Not declared	2
Kotzampassi K (2015) [32]	Greece	164	Probiot	Placebo	Post	14d	L + B + Y	Decreased infectious complications	No	5
Consoli ML (2016) [33]	Brazil	33	Probiot	SC	Pre	7d	Y	No effect	Not declared	3
Komatsu S (2016) [34]	Japan	362	Synbiot	SC	Pre and Post	7–11d & 2–7d	L + B	No effect	Yes	3
Mizuta M (2016) [35]	Japan	60	Probiot	SC	Pre and Post	7–14d & 14d	B	No effect	Not declared	4
Tan CK (2016) [36]	Malaysia	40	Probiot	Placebo	Pre	7d	L + B	No effect	Yes	4
Yang Y (2016) [37]	China	60	Probiot	Placebo	Pre and Post	5d & 7d	L + B + E	No effect	No	5
Flesch AT (2017) [38]	Brazil	91	Synbiot	Placebo	Pre and Post	5d & 14d	L + B	Decreased infectious complications	Not declared	4
Kakaei F (2019) [39]	Iran	99	Probiot	Placebo	Pre	7d	L + B + S	No effect	No	5
Polakowski CB (2019) [40]	Brazil	73	Synbiot	Placebo	Pre	7d.	L + B	Decreased infectious complications	Yes	5
Bajramargic S (2019) [41]	Bosnia and Herzegovina	78	Probiot	SC	Post	1 year	L + B + S	No effect	No	1
Xu Q (2019) [42]	China	60	Probiot	Placebo	Post	7d	B	No effect	No	1
Park IJ (2020) [43]	Korea	59	Probiot	Placebo	Pre and Post	7d & 21d	L + B	No effect	Yes	5
Wang P (2020) [44]	China	51	Probiot	Placebo	Pre and Post	NA	L + B + E	NA	No	5

Pre = preoperatively, Post = postoperatively, d = days, SC = standard care, NA = not available, L = Lactobacillus, B = Bifidobacterium, S = Streptococcus, E = Enterococcus, Y = Yeast.

**Table 2:** Details of the strains [24,25,26,27,28,29,30,31,32,33,34,35,36,37,38,39,40,41,42,43,44].

	Probiotic Strains					Prebiotic		
Authors	*Lactobacillus*	*Bifidobacterium*	*Streptococcus*	*Enterococcus*	Yeast	Oligosaccharides	Oligofructose	Inulin
Reddy BS et al. [24]	X	X	X				X	
Horvat M et al. [25]	X					X		X
Liu Z et al. [26]	X	X						
Mangell B et al. [27]	X							
Zhang JW et al. [28]	X	X		X				
Liu ZH et al. [29]	X	X						
Krebs B et al. [30]	X							X
Sadahiro S et al. [31]		X						
Kotzampassi K et al. [32]	X	X			X			
Consoli ML et al. [33]					X			
Komatsu S et al. [34]	X	X				X		
Mizuta M et al. [35]		X						
Tan CK et al. [36]	X	X						
Yang Y et al. [37]	X	X		X				
Flesch AT et al. [38]	X	X				X	X	
KaKaei F et al. [39]	X	X	X					
Polakowski CB et al. [40]	X	X				X	X	
Bajramargic S et al. [41]	X	X	X					
Xu Q et al. [42]		X						
Park IJ et al. [43]	X	X						
Wang P et al. [44]	X	X		X				

**Table 3:** Summary of findings: Quality of evidence according to GRADE.

Outcome	Effect Size (RR [95%CI])	Heterogeneity (I^2^)	ARR	NNT	Factors of Confidence	Quality of Evidence	Comments
Overall infectious complications	0.59 [0.47–0.75]	15%	−9%	11	⊕ ⊕ ⊕ − ⊕	Moderate	
Surgical site infections	0.70 [0.50–0.95]	0%	−4.40%	23	⊕ − ⊕ − ⊕	Low	No or low heterogeneity.
Pulmonary infections	0.35 [0.20–0.63]	0%	−7%	14	⊕ ⊕ ⊕ − ⊕	Moderate	Significant ARR mainly for overall and non-surgical infectious complications.
Urinary infections	0.41 [0.19–0.87]	0%	−5.70%	17	⊕ ⊕ ⊕ − ⊕	Moderate	The data do not allow any particular timing or duration of probiotic or synbiotic use to be recommended
Anastomotic leak	0.83 [0.47–1.48]	29%	−0.40%	2	⊕ − ⊕ − ⊕	Low	
Wound infection	0.74 [0.53–1.03]	0%	−3.80%	26	⊕ − ⊕ − ⊕	Low	

Factors of confidence are: risk of bias, precision of the effect estimates, consistency of the individual study results, how directly the evidence answers the question of interest, risk of publication or reporting biases, respectively. ARR = absolute risk reduction, NNT = number needed to treat.

**Table 4:** Characteristics and results of published meta-analyses focusing on colorectal surgery and our own meta-analysis [9,10,11,12,13,14,15,16].

First Author	Year of Publication	RCT (n)	Regimen	Overall Infectious Complications (RR [95%CI])	Surgical Site Infections (RR [95%CI])	Pulmonary Infections (RR [95%CI])	Urinary Infections (RR [95%CI])	Subgroups Analyses
He D. [9]	2013	6	probiotics/ synbiotics	0.39 [0.22–0.68]	NA	0.32 [0.11–0.93]	NA	Quality of RCT, publications bias
De Abdrade Calaça PR. [10]	2017	7	probiotics	0.53 [0.40–0.71]	NA	NA	NA	None
Liu PC. [11]	2017	9	probiotics	0.59 [0.43–0.83]	0.67 [0.49–0.93]	0.25 [0.11–0.60]	0.39 [0.16–0.96]	Probiotic formulations
Wu XD. [12]	2017	14	probiotics	NA	0.72 [0.56–0.92]	0.50 [0.29–0.84]	0.50 [0.25–0.98]	Publication bias
Chen C. [13]	2019	6	probiotics	0.31 [0.15–0.64]	0.62 [0.39–0.99]	0.36 [0.18–0.71]	0.26 [0.11–0.60]	None
Ouyang K. [14]	2019	10	probiotics	0.51 [0.38–0.68]	NA	0.56 [0.32–0.98]	0.61 [0.32–1.19]	Publication bias
Amitay EL. [15]	2020	11	probiotics/ synbiotics	0.34 [0.21–0.54]	NA	NA	NA	None
Zeng J. [16]	2021	19	probiotics/ synbiotics	0.37 [0.27–0.53]	0.43 [0.31–0.58]	0.31 [0.18–0.55]	0.41 [0.19–0.87]	Intervention type, strain type, intervention time
Our meta-analysis	2022	21	probiotics/ synbiotics	0.59 [0.47–0.75]	0.70 [0.50–0.95]	0.35 [0.20–0.63]	59 [0.47–0.75]	Intervention type, strain type, intervention time, controls, quality of RCT, competing interests, ethnicity

RCT = randomised controlled trial, RR = Risk ratio, NA = not available.

## Supplementary Materials

The following are available online at https://www.mdpi.com/article/10.3390/nu14153066/s1, Figure S1: Forest plot of pulmonary infections [24,26,28,29,32,36,37,38,39,43], Figure S2: Forest plot of urinary infections [26,29,32,36,37,43], Figure S3: Forest plot of anastomotic leaks [26,28,31,32,34,35,36,37,39,41,43], Figure S4: Forest plot of wound infections [24,25,26,31,34,35,36,37,38,39,42], Figure S5: Forest plot of probiotics [26,27,28,31,32,33,35,36,37] and synbiotics [24,34,40] subgroups, Figure S6: Forest plot of placebo [26,27,28,32,36,37,40] and standard care [24,31,33,34,35] for overall infectious complications, Figure S7: Forest plot of placebo [28,32,36,37,38,39] and standard care [24,31,34,35,41] for surgical site infections, Figure S8: Forest plot of infectious complications according to the Jadad score (≥3) [24,26,27,28,32,33,34,35,36,37,40], Figure S9: Forest plot of overall infectious complications according to ethnicity (Asia [26,28,31,34,35,36,37]; Occident [24,27,32,33,40]), Figure S10: Forest plot of overall infectious complications according to competing interests of authors (No Competing interests [24,27,32,37]; Competing interests [26,34,36,40]; not available [28,31,33,35]).

## References

[1] Magill S.S., O’Leary E., Janelle S.J., Thompson D.L., Dumyati G., Nadle J., Wilson L.E., Kainer M.A., Lynfield R., Greissman S. (2018). Emerging Infections Program Hospital Prevalence Survey Team. Changes in Prevalence of Health Care-Associated Infections in U.S. Hospitals. N. Engl. J. Med..

[2] Fuglestad M.A., Tracey E.L., Leinicke J.A. (2021). Evidence-based Prevention of Surgical Site Infection. Surg. Clin. North Am..

[3] Kiran R.P., El-Gazzaz G.H., Vogel J.D., Remzi F.H. (2010). Laparoscopic approach significantly reduces surgical site infections after colorectal surgery: Data from national surgical quality improvement program. J. Am. Coll. Surg..

[4] Blanc M.C., Slim K., Beyer-Berjot L. (2020). Best practices in bowel preparation for colorectal surgery: A 2020 overview. Expert Rev. Gastroenterol. Hepatol..

[5] Espin Basany E., Solís-Peña A., Pellino G., Kreisler E., Fraccalvieri D., Muinelo-Lorenzo M., Maseda-Díaz O., García-González J.M., Santamaría-Olabarrieta M., Codina-Cazador A. (2020). Preoperative oral antibiotics and surgical-site infections in colon surgery (ORALEV): A multicentre, single-blind, pragmatic, randomised controlled trial. Lancet Gastroenterol. Hepatol..

[6] Hill C., Guarner F., Reid G., Gibson G.R., Merenstein D.J., Pot B., Morelli L., Canani R.B., Flint H.J., Salminen S. (2014). Expert consensus document. The International Scientific Association for Probiotics and Prebiotics consensus statement on the scope and appropriate use of the term probiotic. Nat. Rev. Gastroenterol. Hepatol..

[7] Thomas L.V., Ockhuizen T. (2012). New insights into the impact of the intestinal microbiota on health and disease: A symposium report. Br. J. Nutr..

[8] Komatsu S., Yokoyama Y., Nagino M. (2017). Gut microbiota and bacterial translocation in digestive surgery: The impact of probiotics. Langenbecks Arch. Surg..

[9] He D., Wang H.Y., Feng J.Y., Zhang M.M., Zhou Y., Wu X.T. (2013). Use of pro-/synbiotics as prophylaxis in patients undergoing colorectal resection for cancer: A meta-analysis of randomized controlled trials. Clin. Res. Hepatol. Gastroenterol..

[10] de Andrade Calaça P.R., Pedrosa Bezerra R., Campos Albuquerque W.W., Figueiredo Porto A.L., Holanda Cavalcanti M.T. (2017). Probiotics as a preventive strategy for surgical infection in colorectal cancer patients: A systematic review and meta-analysis of randomized trials. Transl. Gastroenterol. Hepatol..

[11] Liu P.C., Yan Y.K., Ma Y.J., Wang X.W., Geng J., Wang M.C., Wei F.X., Zhang Y.W., Xu X.D., Zhang Y.C. (2017). Probiotics Reduce Postoperative Infections in Patients Undergoing Colorectal Surgery: A Systematic Review and Meta-Analysis. Gastroenterol. Res. Pract..

[12] Wu X.D., Xu W., Liu M.M., Hu K.J., Sun Y.Y., Yang X.F., Zhu G.Q., Wang Z.W., Huang W. (2018). Efficacy of prophylactic probiotics in combination with antibiotics versus antibiotics alone for colorectal surgery: A meta-analysis of randomized controlled trials. J. Surg. Oncol..

[13] Chen C., Wen T., Zhao Q. (2020). Probiotics Used for Postoperative Infections in Patients Undergoing Colorectal Cancer Surgery. Biomed. Res. Int..

[14] Ouyang X., Li Q., Shi M., Niu D., Song W., Nian Q., Li X., Ding Z., Ai X., Wang J. (2019). Probiotics for preventing postoperative infection in colorectal cancer patients: A systematic review and meta-analysis. Int. J. Colorectal. Dis..

[15] Amitay E.L., Carr P.R., Gies A., Laetsch D.C., Brenner H. (2020). Probiotic/Synbiotic Treatment and Postoperative Complications in Colorectal Cancer Patients: Systematic Review and Meta-analysis of Randomized Controlled Trials. Clin. Transl. Gastroenterol..

[16] Zeng J., Ji Y., Liang B., Zhang G., Chen D., Zhu M., Wu S., Kuang W. (2021). The effect of pro/synbiotics on postoperative infections in colorectal cancer patients: A systematic review and meta-analysis. Complement. Ther. Clin. Pract..

[17] Slim K., Marquillier T. (2021). Umbrella reviews: A new tool to synthesize scientific evidence in surgery. J. Visc. Surg..

[18] Page M.J., McKenzie J.E., Bossuyt P.M., Boutron I., Hoffmann T.C., Mulrow C.D., Shamseer L., Tetzlaff J.M., Akl E.A., Brennan S.E. (2021). The PRISMA 2020 statement: An updated guideline for reporting systematic reviews. BMJ.

[19] Jadad A.R., Moore R.A., Carroll D., Jenkinson C., Reynolds D.J., Gavaghan D.J., McQuay H.J. (1996). Assessing the quality of reports of randomized clinical trials: Is blinding necessary?. Control. Clin. Trials..

[20] Guyatt G.H., Oxman A.D., Vist G.E., Kunz R., Falck-Ytter Y., Alonso-Coello P., Schünemann H.J., GRADE Working Group (2008). GRADE: An emerging consensus on rating quality of evidence and strength of recommendations. BMJ.

[21] van Aert R.C.M., Jackson D. (2018). Multistep estimators of the between-study variance: The relationship with the Paule-Mandel estimator. Stat. Med..

[22] Michael B., Hedges L.V., Higgins J.P.T., Rothstein H. (2009). Introduction to Meta-Analysis.

[23] Balduzzi S., Rücker G., Schwarzer G. (2019). How to perform a meta-analysis with R: A practical tutorial. Evid. Based Ment. Health.

[24] Reddy B.S., Macfie J., Gatt M., Larsen C.N., Jensen S.S., Leser T.D. (2007). Randomized clinical trial of effect of synbiotics, neomycin and mechanical bowel preparation on intestinal barrier function in patients undergoing colectomy. Br. J. Surg..

[25] Horvat M., Krebs B., Potrc S., Ivanecz A., Kompan L. (2010). Preoperative synbiotic bowel conditioning for elective colorectal surgery. Wien. Klin. Wochenschr..

[26] Liu Z., Qin H., Yang Z., Xia Y., Liu W., Yang J., Jiang Y., Zhang H., Yang Z., Wang Y. (2011). Randomised clinical trial: The effects of perioperative probiotic treatment on barrier function and post-operative infectious complications in colorectal cancer surgery-A double-blind study. Aliment. Pharmacol. Ther..

[27] Mangell P., Thorlacius H., Syk I., Ahrné S., Molin G., Olsson C., Jeppsson B. (2012). Lactobacillus plantarum 299v does not reduce enteric bacteria or bacterial translocation in patients undergoing colon resection. Dig. Dis. Sci..

[28] Zhang J.W., Du P., Gao J., Yang B.R., Fang W.J., Ying C.M. (2012). Preoperative probiotics decrease postoperative infectious complications of colorectal cancer. Am. J. Med. Sci..

[29] Liu Z.H., Huang M.J., Zhang X.W., Wang L., Huang N.Q., Peng H., Lan P., Peng J.S., Yang Z., Xia Y. (2013). The effects of perioperative probiotic treatment on serum zonulin concentration and subsequent postoperative infectious complications after colorectal cancer surgery: A double-center and double-blind randomized clinical trial. Am. J. Clin. Nutr..

[30] Krebs B., Horvat M., Golle A., Krznaric Z., Papeš D., Augustin G., Arslani N., Potrč S. (2013). A randomized clinical trial of synbiotic treatment before colorectal cancer surgery. Am. Surg..

[31] Sadahiro S., Suzuki T., Tanaka A., Okada K., Kamata H., Ozaki T., Koga Y. (2014). Comparison between oral antibiotics and probiotics as bowel preparation for elective colon cancer surgery to prevent infection: Prospective randomized trial. Surgery.

[32] Kotzampassi K., Stavrou G., Damoraki G., Georgitsi M., Basdanis G., Tsaousi G., Giamarellos-Bourboulis E.J. (2015). A Four-Probiotics Regimen Reduces Postoperative Complications After Colorectal Surgery: A Randomized, Double-Blind, Placebo-Controlled Study. World J. Surg..

[33] Consoli M.L., da Silva R.S., Nicoli J.R., Bruña-Romero O., da Silva R.G., de Vasconcelos Generoso S., Correia M.I. (2016). Randomized Clinical Trial: Impact of Oral Administration of Saccharomyces boulardii on Gene Expression of Intestinal Cytokines in Patients Undergoing Colon Resection. JPEN J. Parenter. Enteral Nutr..

[34] Komatsu S., Sakamoto E., Norimizu S., Shingu Y., Asahara T., Nomoto K., Nagino M. (2016). Efficacy of perioperative synbiotics treatment for the prevention of surgical site infection after laparoscopic colorectal surgery: A randomized controlled trial. Surg. Today.

[35] Mizuta M., Endo I., Yamamoto S., Inokawa H., Kubo M., Udaka T., Sogabe O., Maeda H., Shirakawa K., Okazaki E. (2016). Perioperative supplementation with bifidobacteria improves postoperative nutritional recovery, inflammatory response, and fecal microbiota in patients undergoing colorectal surgery: A prospective, randomized clinical trial. Biosci. Microbiota Food Health.

[36] Tan C.K., Said S., Rajandram R., Wang Z., Roslani A.C., Chin K.F. (2016). Pre-surgical Administration of Microbial Cell Preparation in Colorectal Cancer Patients: A Randomized Controlled Trial. World J. Surg..

[37] Yang Y., Xia Y., Chen H., Hong L., Feng J., Yang J., Yang Z., Shi C., Wu W., Gao R. (2016). The effect pf perioperative probiotics treatment for colorectal cancer: Short-term outcomes of a randomized controlled trial. Oncotarget.

[38] Flesch A.T., Tonial S.T., DE Carvalho Contu P., Damin D.C. (2017). Perioperative synbiotics administration decreases postoperative infections in patients with colorectal cancer: A randomized, double-blind clinical trial. Rev. Col. Bras. Cir..

[39] Kakaei F., Shahrasbi M., Asvadi Kermani T., Taheri S., Tarvirdizade K. (2016). Assessment of probiotic effects on colorectal surgery complications: A double blind, randomized clinical trial. Biomed. Res. Ther..

[40] Polakowski C.B., Kato M., Preti V.B., Schieferdecker M.E.M., Ligocki Campos A.C. (2019). Impact of the preoperative use of synbiotics in colorectal cancer patients: A prospective, randomized, double-blind, placebo-controlled study. Nutrition.

[41] Bajramagic S., Hodzic E., Mulabdic A., Holjan S., Smajlovic S.V., Rovcanin A. (2019). Usage of Probiotics and its Clinical Significance at Surgically Treated Patients Sufferig from Colorectal Carcinoma. Med. Arch..

[42] Xu Q., Xu P., Cen Y., Li W. (2019). Effects of preoperative oral administration of glucose solution combined with postoperative probiotics on inflammation and intestinal barrier function in patients after colorectal cancer surgery. Oncol. Lett..

[43] Park I.J., Lee J.H., Kye B.H., Oh H.K., Cho Y.B., Kim Y.T., Kim J.Y., Sung N.Y., Kang S.B., Seo J.M. (2020). Effects of PrObiotics on the Symptoms and Surgical ouTComes after Anterior REsection of Colon Cancer (POSTCARE): A Randomized, double-blind, placebo-controlled trial. J. Clin. Med..

[44] Wang P., Yin X., Chen G., Li L., Le Y., Xie Z., Ouyang W., Tong J. (2021). Perioperative probiotic treatment decreased the incidence of postoperative cognitive impairment in elderly patients following non-cardiac surgery: A randomised double-blind and placebo-controlled trial. Clin. Nutr..

[45] Chen Y., Qi A., Teng D., Li S., Yan Y., Hu S., Du X. (2022). Probiotics and synbiotics for preventing postoperative infectious complications in colorectal cancer patients: A systematic review and meta-analysis. Tech. Coloproctol..

[46] Chowdhury A.H., Adiamah A., Kushairi A., Varadhan K.K., Krznaric Z., Kulkarni A.D., Neal K.R., Lobo D.N. (2020). Perioperative probiotics or synbiotics in adults undergoing elective abdominal surgery: A systematic review and meta-analysis of randomized controlled trials. Ann. Surg..

[47] Dang A.T., Marsland B.J. (2019). Microbes, metabolites, and the gut-lung axis. Mucosal Immunol..

[48] Neugent M.L., Hulyalkar N.V., Nguyen V.H., Zimmern P.E., De Nisco N.J. (2020). Advances in Understanding the Human Urinary Microbiome and Its Potential Role in Urinary Tract Infection. mBio.

[49] Slim K., Theissen A. (2020). Enhanced recovery after elective surgery. A revolution that reduces post-operative morbidity and mortality. J. Visc. Surg..

[50] Ljungqvist O., de Boer H.D., Balfour A., Fawcett W.J., Lobo D.N., Nelson G., Scott M.J., Wainwright T.W., Demartines N. (2021). Opportunities and Challenges for the Next Phase of Enhanced Recovery After Surgery: A Review. JAMA Surg..

