# Supplementary material for: Probiotics/Synbiotics to Reduce Infectious Complications after Colorectal Surgery: A Systematic Review and Meta-Analysis of Randomised Controlled Trials
Source: Nutrients. 2022 Jul 26;14(15):3066. doi: 10.3390/nu14153066 (PMC9332115; doi:10.3390/nu14153066)
Supplement: Supplementary file 1 [file nutrients-14-03066-s001.zip › nutrients-1782047-supplementary.pdf]

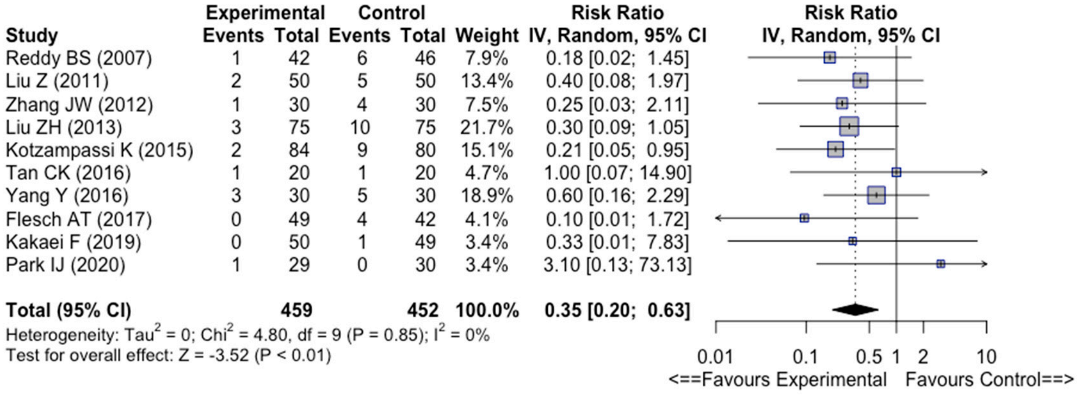

Figure S1: Forest plot of pulmonary infections [24,26,28,29,32,36–39, 43]

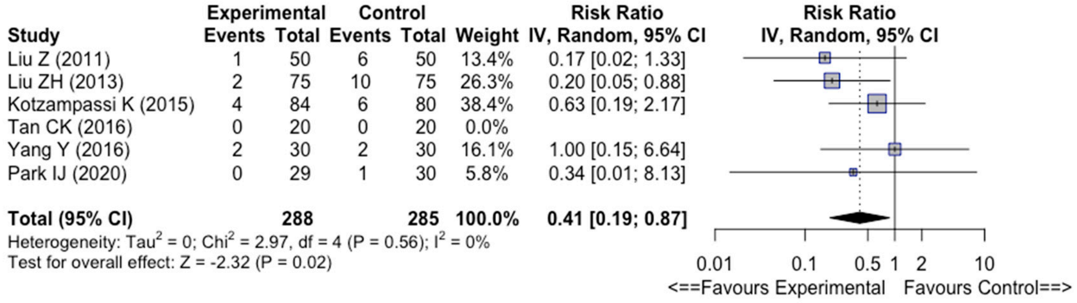

Figure S2: Forest plot of urinary infections [26,29,32,36,37,43]

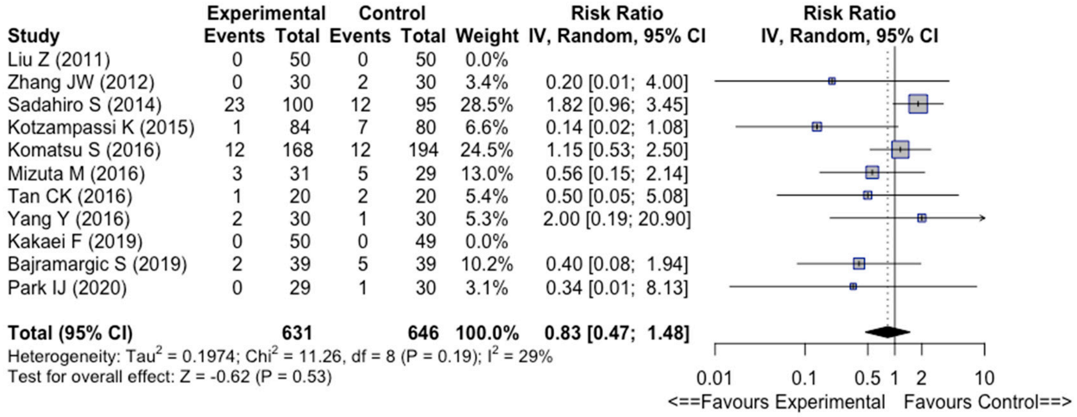

Figure S3: Forest plot of anastomotic leaks [26,28,31,32,34–37,39,41,43]

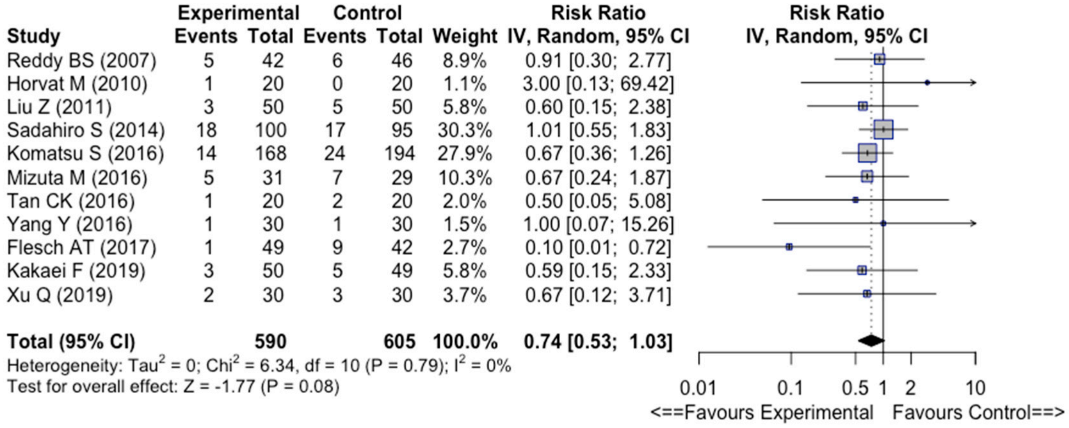

Figure S4: Forest plot of wound infections [24–26,31,34–39,42]

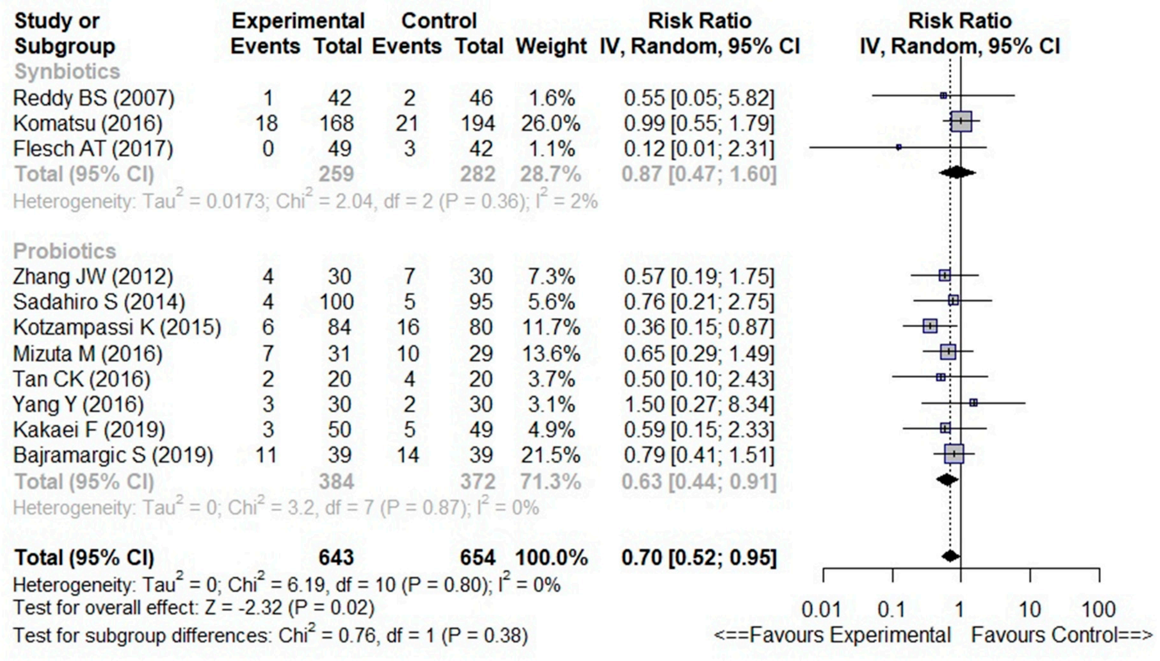

Figure S5: Forest plot of probiotics [26–28,31–33,35–37] and synbiotics [24,34,40] subgroup

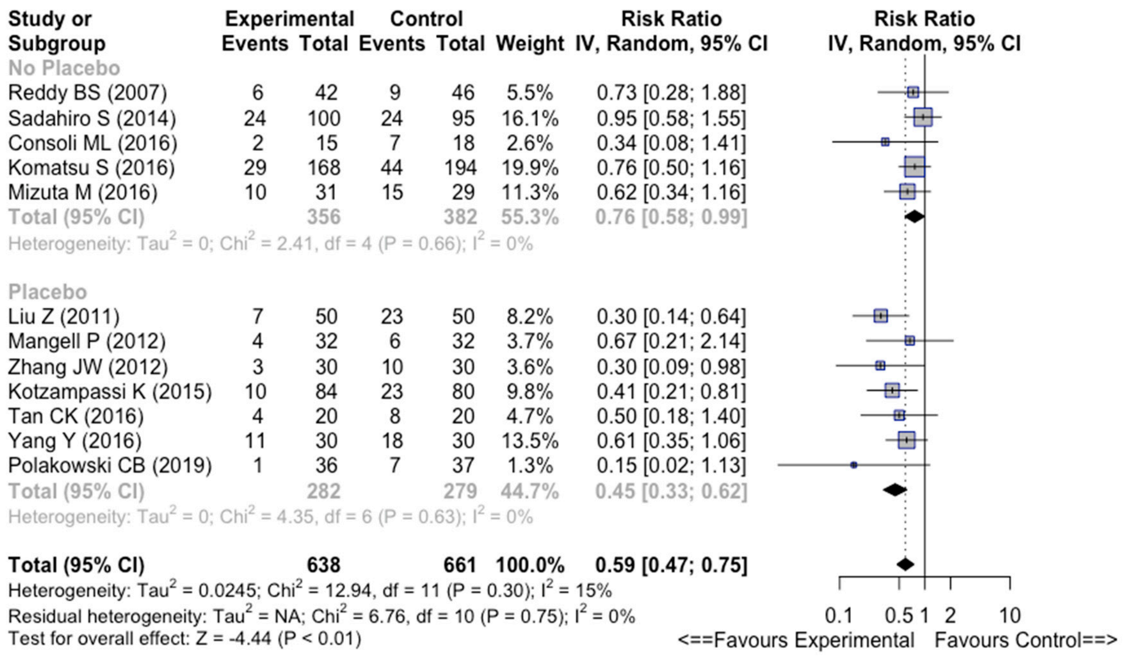

Figure S6: Forest plot of placebo [26–28,32,36,37,40] and standard care [24,31,33–35] for overall infectious complications

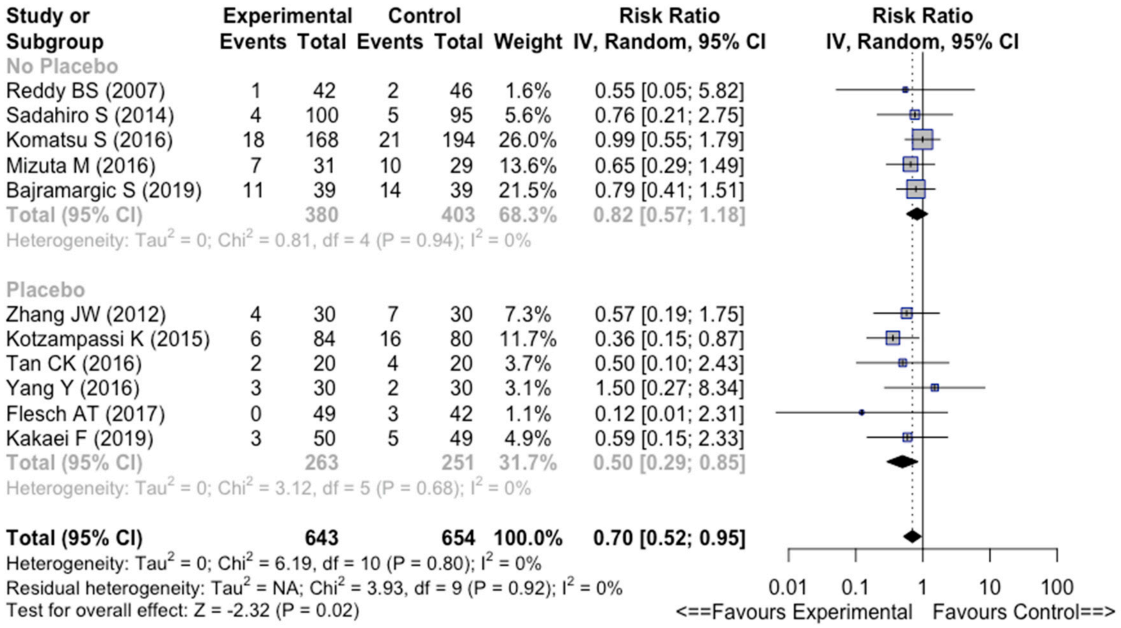

Figure S7: Forest plot of placebo [28,32,36–39] and standard care [24,31,34,35,41] for surgical site infections

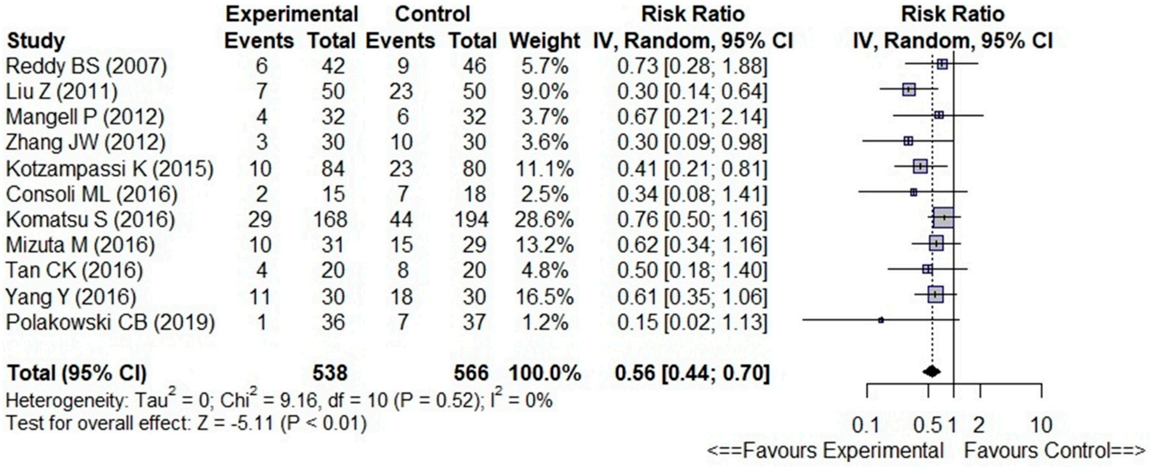

Figure S8: Forest plot of infectious complications according to the Jadad score ≥3 [24,26-28,32–37,40]

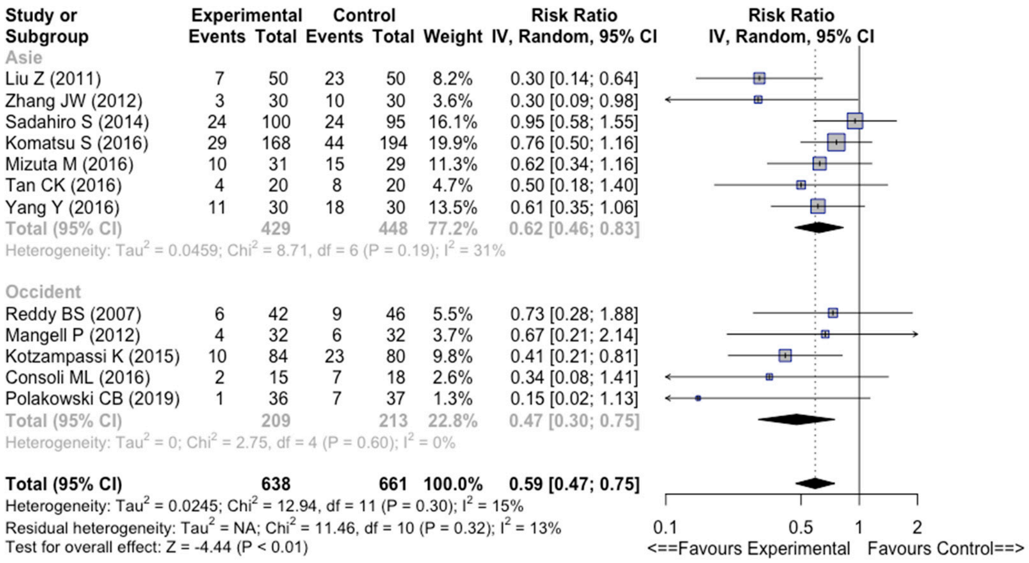

Figure S9: Forest plot of overall infectious complications according to ethnicity (Asia [26,28,31,34–37]; Occident [24,27,32,33,40])

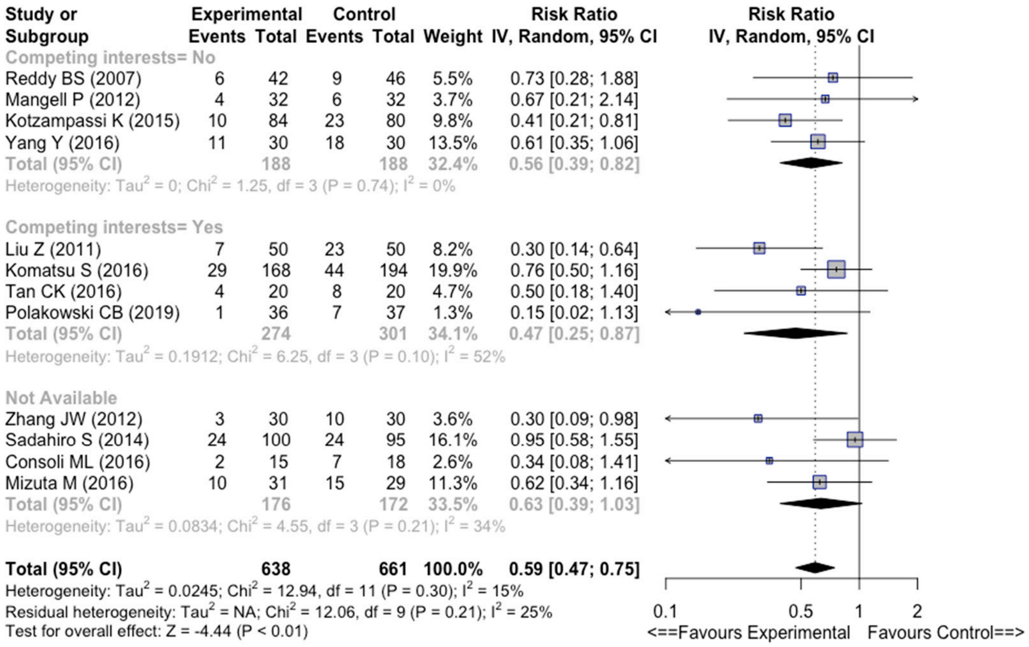

Figure S10: Forest plot of overall infectious complications according to competing interest of authors (No Competing interests [24,27,32,37] ; Competing interests [26,34,36,40] ; not available [28,31,33,35])
